# Cyclin-dependent kinases in *C. elegans*

**DOI:** 10.1186/1747-1028-1-6

**Published:** 2006-05-12

**Authors:** Mike Boxem

**Affiliations:** 1 Massachusetts General Hospital Cancer Center, Building 149, 13th Street, Charlestown, MA 02129, USA; 2 Center for Cancer Systems Biology (CCSB) and Department of Cancer Biology, Dana-Farber Cancer Institute, 44 Binney St, Boston MA 02115, USA

## Abstract

Cell division is an inherent part of organismal development, and defects in this process can lead to developmental abnormalities as well as cancerous growth. In past decades, much of the basic cell-cycle machinery has been identified, and a major challenge in coming years will be to understand the complex interplay between cell division and multicellular development. Inevitably, this requires the use of more complex multicellular model systems. The small nematode *Caenorhabditis elegans *is an excellent model system to study the regulation of cell division in a multicellular organism, and is poised to make important contributions to this field. The past decade has already seen a surge in cell-cycle research in *C. elegans*, yielding information on the function of many basic cell-cycle regulators, and making inroads into the developmental control of cell division. This review focuses on the *in vivo *roles of cyclin-dependent kinases in *C. elegans*, and highlights novel findings implicating CDKs in coupling development to cell-cycle progression.

## Background

*Caenorhabditis elegans *is a small, soil-dwelling nematode with a simple body plan formed by 959 somatic cells in adult hermaphrodites and 1031 somatic cells in adult males. Under laboratory conditions, *C. elegans *develops from a one cell embryo to a fertile adult in 3–5 days, depending on culture temperature. The life cycle of *C. elegans *consists of an embryonic stage, 4 larval stages (L1–L4), and an adult stage. Embryonic divisions generate 558 nuclei (a number of *C. elegans *tissues including the intestine and hypodermis are syncytial, hence "nuclei" more accurately describes the lineage) [1]). Nearly all embryonic cell-divisions are completed in the first half of embryogenesis [1], and these early cleavages are largely under the control of proteins and mRNA deposited in the oocyte by the mother.

During the larval stages, 53 somatic blast cells will undergo further divisions to generate the final 959 or 1031 somatic nuclei [2], while no somatic divisions take place in the adult stage. Most larval divisions are normal mitotic divisions, although the intestinal and hypodermal nuclei also undergo several rounds of endoreplication [3]. The germline, which contains ~2000 germ cells in adults, is populated by divisions of an additional 2 blast cells [4].

### Studying cell division in *C. elegans*

**Figure 1:**
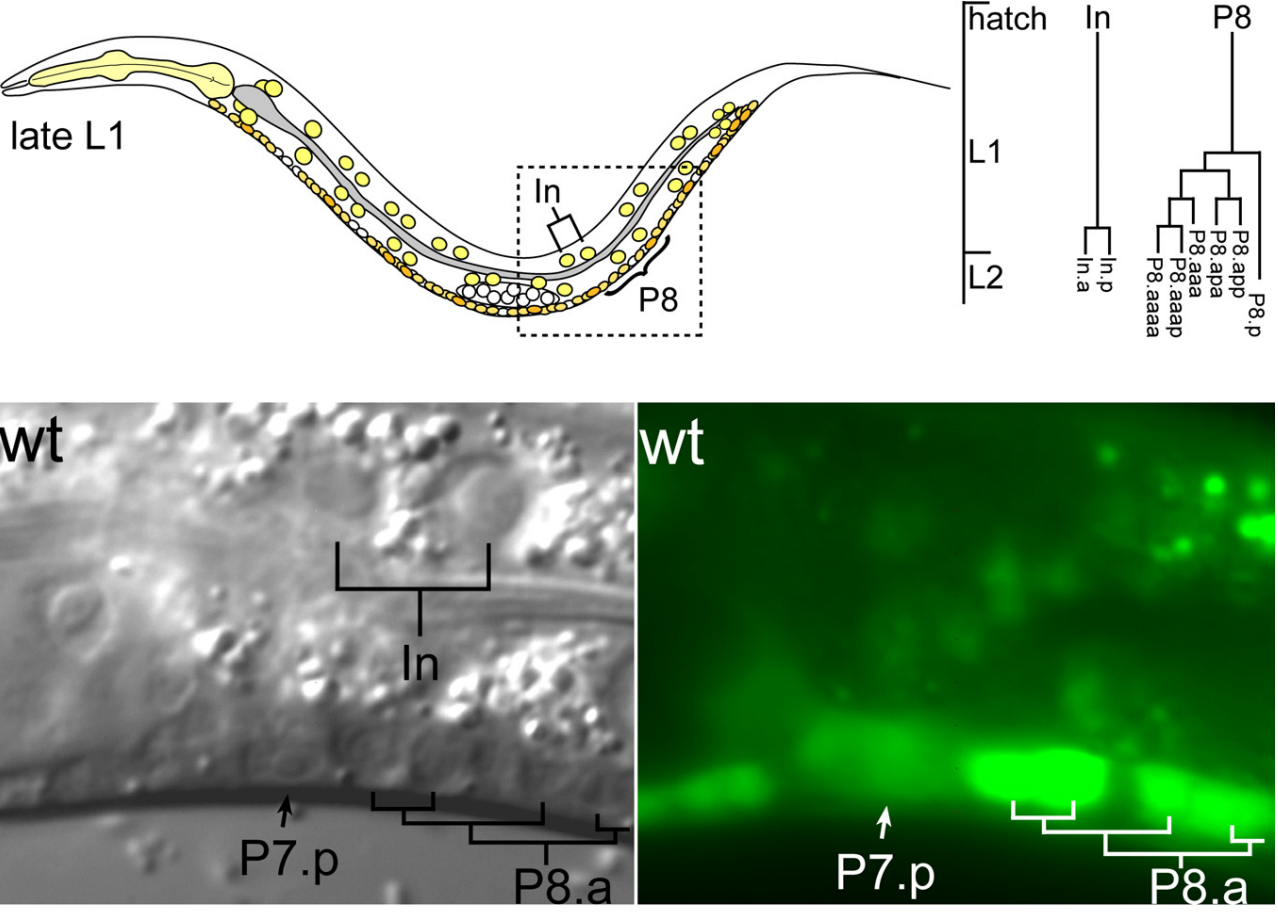
**Using GFP markers to aid cell-cycle studies**. (Top) Schematic drawing of a late L1 larva with intestinal and ventral cord cells indicated. The cell lineage for an intestinal nucleus (In) and ventral cord precursor cell (P8) is drawn to the right. (Bottom) Nomarski DIC and GFP fluorescence image from a late L1 larva carrying a transgene expressing GFP under control of ribonucleotide reductase regulatory sequences (*P*_*rnr*_*::GFP*). Imaged area corresponds approximately to the boxed area in the schematic drawing. GFP expression correlates with progression through S-phase, and can thus be used to distinguish a G1 arrest from a later arrest. In this image, *P*_*rnr*_*::GFP *is expressed in the descendants of P8.

*C. elegans *is a unique multicellular model system for studies of cell division because of its nearly invariable developmental program and cell lineage. The relative timing of divisions, the orientation of division axes, and the final cell fates, are all highly reproducible, and the entire cell lineage from the one cell embryo to the adult has been described [1,2]. Furthermore, cell divisions can be observed *in vivo *under Nomarski DIC microscopy, and reporter genes such as green fluorescent protein (GFP) can be used to mark specific cell-cycle phases or cell lineages in live animals (figure 1). Thus, defects in the pattern of cell divisions ran readily be detected.

**Figure 2:**
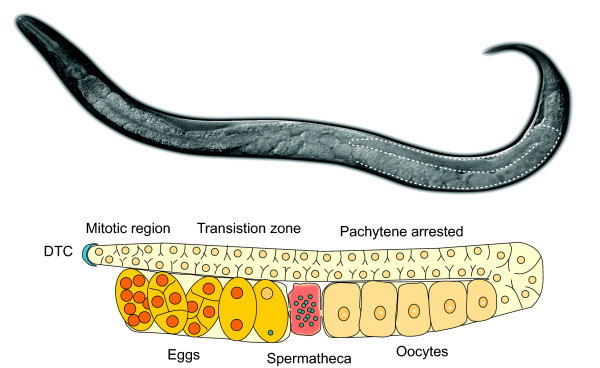
**The *C. elegans *reproductive system**. (Top) Nomarski DIC image of an adult hermaphrodite. The hermaphrodite reproductive system consists of two U-shaped gonad arms, in which germ cells develop in an assembly-line fashion from mitotic divisions at the distal end to ovulation and fertilization at the proximal end [99]. Dotted lines surround the posterior gonad arm. (Bottom) Schematic drawing of one gonad arm. Germ nuclei are generated by mitotic divisions in response to a signal from the Distal Tip Cell (DTC) at the distal end of each gonad arm. As the nuclei move away from the DTCs they initiate meiosis and arrest at the pachytene stage of meiosis I. Around the time the nuclei reach the bend in the gonadal arm, oogenesis is initiated. The germ nuclei become fully enclosed by a plasma membrane, and the resulting oocyte grows dramatically in size. The germ cells exit pachytene, and progress through diplotene arresting for a second time in diakinesis, the final stage of meiotic prophase. The oocytes proceed in single file through the gonad arm, with the most mature oocyte present directly adjacent to the spermatheca.

The different phases of *C. elegans *development each offer distinct advantages and disadvantages for cell-division studies. For example, early embryonic cells are well suited to study subcellular structures such as the spindle apparatus, and the physiology of adult hermaphrodites lends itself exceptionally well to studies of meiotic maturation (figure 2). In contrast, checkpoint controls and global developmental control of cell division are best studied during larval development, as these processes are mostly absent during embryonic divisions, and the embryo is therefore unsuitable for such studies.

The ability to use RNA interference (RNAi) as well as mutant alleles offers a powerful toolkit to examine gene function at different stages in the *C. elegans *life cycle. Animals lacking both copies of critical cell-cycle regulators are necessarily derived from heterozygous parents. Since most embryonic cell-divisions are completed on maternally contributed stores of protein and mRNA, these mutants frequently display phenotypes only during larval development. In contrast, RNAi affects both maternal and embryonic mRNAs, and can be used to examine the embryonic roles of cell-cycle regulators. Finally, RNAi by feeding, in which dsRNA is delivered by way of the bacteria *C. elegans *feeds on, allows for application of RNAi at different stages of postembryonic development, and can be used to further increase the range of developmental stages at which gene function can be examined.

### Eukaryotic cell cycle regulation

**Figure 3:**
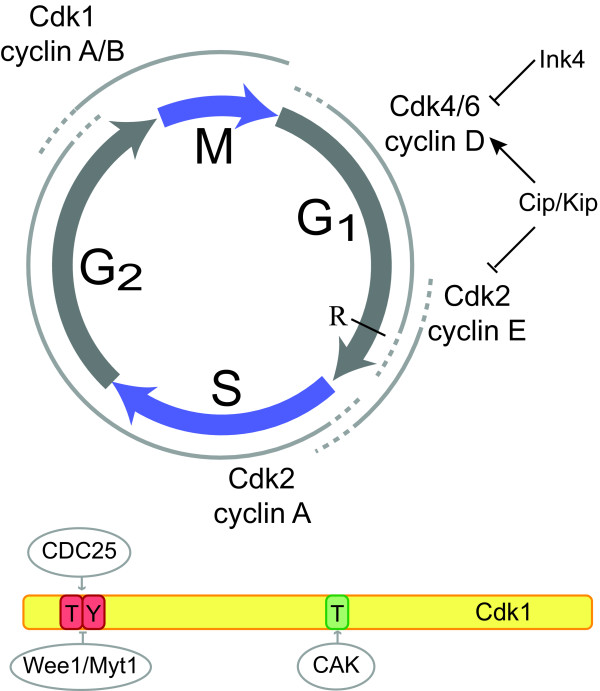
**Cell cycle control in higher eukaryotes**. (Top) Cyclin-CDK complexes in higher eukaryotes, and their approximate times of activity during the cell cycle. For clarity, extended cyclin families are indicated only by their class name (i.e. cyclin D rather than cyclin D1, D2, D3). The two known families of CDK inhibitors are also indicated. R indicates the Restriction point, beyond which cells do not required growth-factor signaling to complete cell division. (Bottom) Control of Cdk1 activity by phosphorylation. Phosphorylation of a conserved Thr residue by CAK is required for full activation. Phosphorylation of Tyr15 by Wee1 or both Thr14 and Tyr15 by Myt1 blocks Cdk1 activity, and is counteracted by members of the Cdc25 family of phosphatases.

Progression through the eukaryotic cell cycle is controlled by the activities of cyclin-dependent kinases (CDKs), the founding members of which are the highly homologous 34kDa proteins encoded by the *cdc2 *gene in the fission yeast *Schizosaccharomyces pombe *and the *CDC28 *gene in the budding yeast *Schizosaccharomyces cerevisiae *[5-7]. Both yeasts use a single CDK combined with different cyclin subunits to control progression through the division cycle. In higher eukaryotes, the transitions between successive phases of the cell cycle are controlled by the activities of multiple CDKs in combination with different families of cyclins (figure 3). CDK activity is tightly controlled by a combination of mechanisms. First, the levels of available cyclin subunits are regulated through protein synthesis and protein degradation [8,9]. Second, phosphorylation of several conserved residues can increase or decrease the activity of CDKs (figure 3). To achieve full activity of Cdk1, Cdk2, Cdk4 and Cdk6, a conserved Threonine residue (T160 in human Cdk2) needs to be phosphorylated by the CDK-activating kinase (CAK) [10]. Multiple CDKs are phosphorylated on inhibitory residues near the N-terminus (a single Tyrosine in Cdk4 and Cdk6 and adjacent Threonine and Tyrosine residues in Cdk1 and Cdk2) [11-16]. Cdk1 and Cdk2 are phosphorylated by Wee1 and Myt1 kinases [14-16], while the kinase(s) responsible for phosphorylating Cdk4 and Cdk6 are yet to be discovered. Dephosphorylation of these residues is performed by the Cdc25 family of phosphatases. Whereas phosphorylation and dephosphorylation of Thr14/Tyr15 is critical for control of Cdk1 activity [11], the importance of inhibitory phosphorylation in the other CDKs is less well understood. For example, phosphorylation on the corresponding sites in Cdk2 may have no or limited effect on its activity [17,18].

Finally, Cyclin/CDK activity can be blocked by CDK inhibitors of the Ink4 and Cip/Kip families [19]. Paradoxically, Cip/Kip family members may also be required for the assembly of Cdk4 and Cdk6 with D-type cyclins [20,21]. Together, these mechanisms ensure a tightly regulated peak of CDK activity at the appropriate time.

### *C. elegans *cell-cycle regulators

With the notable exception of the Ink4 family, all major cell-cycle regulators found in mammals are also present in *C. elegans *([22,23] and table 1). The *C. elegans *genome encodes 14 members of the CDK family [23]. Of these, 3 are core cell-cycle regulators, while 2 have largely cell-cycle independent roles. The functions of the remaining 9 CDKs remain unclear to date.

**Table 1:** CDKs and regulatory proteins encoded by the *C. elegans *genome

**Protein**	***C. elegans *homolog**		**Function**
	Protein name	Cosmid name	
**CDKs**			

CDK1	CDK-1	T05G5.3	Entry into mitosis^(e) ^[25]
CDK2	-	K03E5.3	S phase entry/progression^(p)^
CDK4/CDK6	CDK-4	F18H3.5	G1/S progression^(e) ^[64, 100]
CDK5	CDK-5	T27E9.3	Neuronal development/functioning^(p)^
CDK7	CDK-7	Y39G10AL.3	CDK activating kinase, RNA pol II phosphorylation^(e) ^[81]
CDK8	CDK-8	F39H11.3	Transcriptional regulation^(p)^
CDK9	CDK-9	H25P06.2	RNA pol II phosphorylation^(e) ^[82]
**Cyclins**			

Cyclin A	CYA-1	ZK507.6	CDK-1/CDK-2 partner^(p)^
	CYA-2	F59H6.7	CDK-1/CDK-2 partner^(p)^
Cyclin B	CYB-1	ZC168.4	CDK-1 partner^(e) ^[31]
	CYB-2.1	Y43E12A.1	CDK-1 partner^(p)^
	CYB-2.2	H31G24.4	CDK-1 partner^(p)^
Cyclin B3	CYB-3	T06E6.2	CDK-1 partner^(e) ^[31]
Cyclin C	CIC-1	H14E04.5	CDK-8 partner^(p)^
Cyclin D	CYD-1	Y38F1A.5	CDK-4 partner^(e) ^[64]
Cyclin E	CYE-1	C37A2.4	CDK-2 partner^(p)^
Cyclin H	CYH-1	Y49F6B.1	CDK-7 partner^(p)^
Cyclin T	CIT-1.1	F44B9.4	CDK-9 partner^(p)^
	CIT-1.2	F44B9.3	CDK-9 partner^(p)^
p35	CDKA-1	T23F11.3	CDK-5 activating subunit^(p)^
**Other**			

Cip/Kip	CKI-1	T05A6.1	Negative regulator of G1 progression, likely through inhibition of CDK-2^(e) ^[70]
	CKI-2	T05A6.2	Unknown
Wee1/Myt1	WEE-1.1	F35H8.7	Negative regulator of CDK-1^(p)^
	WEE-1.3	Y53C12A.1	Negative regulation of meiotic progression, likely through CDK-1 phosphorylation^(e) ^[32]
Cdc25	CDC-25.1	K06A5.7	Dephosphorylation of inhibitory CDK residues^(*p*)^
	CDC-25.2	F16B4.8	Dephosphorylation of inhibitory CDK residues^(p)^
	CDC-25.3	ZK637.11	Dephosphorylation of inhibitory CDK residues^(p)^
	CDC-25.4	R05H5.2	Dephosphorylation of inhibitory CDK residues^(p)^
CKS1	DOM-6	C09G4.3	Required for exit from meiosis and mitosis^(e) ^[97]

*C. elegans *homologs of mammalian CDKs for with established functions, their predicted cyclin partners, and several key regulators of CDK activity.

^(e) ^function experimentally determined in *C. elegans*

^(p) ^function predicted based on homologs in other organisms

## Cell-cycle regulatory CDKs in *C. elegans*

### CDK-1

The first cyclin dependent kinase studied in *C. elegans *is encoded by the *cdk-1 *gene (originally named *ncc-1 *for Nematode Cell Cycle) [24]. *cdk-1 *mutant animals fail to undergo any larval cell divisions, while inactivation of *cdk-1 *by RNAi blocks mitotic and meiotic divisions in the germline of the injected animal, as well as mitotic division of the one cell embryo [25]. Experiments using a temperature sensitive *cdk-1 *allele demonstrated a critical role in later larval divisions as well [25]. Thus, CDK-1 appears to be essential for all *C. elegans *cell divisions.

*cdk-1 *mutant cells fail to undergo mitosis, but do express a common S-phase marker: GFP under control of ribonucleotide reductase regulatory sequences (*P*_*rnr*_*::GFP*) (figure 1). In addition, they incorporate the nucleotide analog BrdU into their DNA [25]. Furthermore, the intestinal nuclei are still able to undergo their normal 4 rounds of endoreplication in *cdk-1 *mutant animals [25]. Thus, like mammalian Cdk1, CDK-1 is specifically required for mitosis. Importantly, these experiments demonstrated that *C. elegans *uses specific CDKs to drive progression through the cell cycle, similar to higher eukaryotes and vertebrates.

Mammalian Cdk1 partners with A- and B-type cyclins. The *C. elegans *genome encodes 2 predicted cyclin A homologs (CYA-1 and CYA-2), three B-type cyclins (CYB-1, CYB-2.1 and CYB-2.2), and one B3 type cyclin (CYB-3) [26]. Genome wide RNAi screens indicate that all but CYA-2 are required for embryonic cell divisions [27-30], and CYB-1 and CYB-3 were shown to bind to CDK-1 *in vivo *[31]. A detailed characterization of the individual functions of these cyclins is still lacking, however.

#### Regulation of CDK-1 in meiotic maturation

In addition to its role in regulating mitotic cell divisions, Cdk1 plays a key role in the maturation of developing oocytes of higher eukaryotes as the kinase component of Maturation Promoting Factor (MPF). As in other organisms, *C. elegans *CDK-1 is critical for meiotic maturation and completion of meiotic divisions [25,32]. The mechanisms that activate MPF when maturation is triggered are not fully known in any system, and vary between organisms. For example, *Xenopus laevis *oocytes contain a stockpile of pre-formed Cdk1/cyclin B complexes (pre-MPF) that is activated by dephosphorylation of Thr14 and Tyr15, while other amphibians contain monomeric Cdk1 and depend on synthesis of B-type cyclins for MPF activation [33,34].

A recent study examining the role of the *C. elegans *Myt1 homolog WEE-1.3 indicates that in *C. elegans*, pre-formed cyclin B-CDK-1 complexes are present and kept inactive through negative phosphorylation by WEE-1.3 [32]. In wild-type animals, only the most proximal oocyte initiates maturation in response to a component secreted by sperm termed major sperm protein (MSP) [35]. Loss of *wee-1.3 *results in precocious oocyte maturation, which can be blocked by the inactivation of *cdk-1 *or all 4 *C. elegans *B-type cyclins together. Furthermore, in the absence of *wee-1.3*, inhibitory phosphorylation of CDK-1 is not observed [36]. Thus, the precocious oocyte maturation in *wee-1.3(RNAi) *oocytes is likely the result of an inability to keep cyclin B-CDK-1 complexes inactive in the more distal oocytes.

Interestingly, oocyte maturation is not initiated when *wee-1.3 *is inactivated in animals lacking sperm and thus MSP [32], indicating that sperm is not only required for maturation of the most proximal oocyte, but also for the generation of cyclin B-CDK-1 complexes in more distal oocytes. How then are all but the most proximal oocyte blocked from initiating maturation? It seems likely that mechanisms in addition to MSP remain to be discovered, as MSP and its receptor VAB-1 are detectable around at least 3 oocytes [37], and MAPK phosphorylation, an indicator of MSP activity, is also found in multiple oocytes [35].

Another level of control over Cdk1 activity is degradation of B-type cyclins at the end of mitosis, which is triggered by the anaphase-promoting complex/cyclosome (APC/C) [9]. The dependence upon the APC/C in meiosis varies between organisms. For example, in *S. Cerevisiae *two rounds of APC/C activity sequentially degrade meiosis specific cohesin complexes containing Rec8 [38-40], while APC/C is dispensable for meiosis I in *Xenopus *oocytes [41,42].

In *C. elegans*, inactivation of the APC/C blocks the metaphase to anaphase transition in meiosis I in oocytes and sperm [43-48]. Although the role of cyclin-B degradation has not been directly studied, protein levels of the B-type cyclins CYB-1 and CYB-3 are high in maturing oocytes, and quickly drop in meiosis I [49,50]. Furthermore, inactivation of *apc-11 *stabilizes CYB-1 levels [49]. It is likely therefore that degradation of B-type cyclins by the APC/C plays a role in progression through meiosis I in *C. elegans*.

Interestingly, *C. elegans *APC/C does not appear to play a key role in progression through meiosis II [45]. Consistent with this observation, two groups recently identified a novel ubiquitin ligase complex to be required for exit from meiosis II [49,50]. This E3 ubiquitin ligase complex contains the cullin CUL-2, ELC-1 elongin C, RBX-1 Rbx, and the novel component ZYG-11. The B-type cyclins CYB-1 and CYB-3 appear to be regulated by the CUL-2 based E3, as their expression levels are stabilized upon loss of E3 activity. CYB-1 and CYB-3 appear to have partially non redundant functions in meiosis II, as loss of *cyb-3 *rescues the duration of anaphase II (but not metaphase II) in a *zyg-11 *mutant [50], while loss of *cyb-1 *partially restores the duration of metaphase II in a *cul-2 *mutant [49]. These experiments indicate that an alternative mechanism of B-type cyclin degradation is used for progression through meiosis II in *C. elegans*.

The functions of the APC/C and CUL-2/ZYG-11 complexes may not be entirely restricted to one meiotic phase, as *zyg-11 *and *cul-2 *do enhance weak APC/C alleles, pointing to a partially redundant function in meiosis I. Further experiments will be needed to determine the exact roles of cyclin degradation in meiosis, and to determine how REC-8 is degraded in meiosis II.

### CDK-2

Although no definitive *C. elegans *Cdk2 homolog has been identified, the most likely candidate is encoded by the K03E5.3 gene, whose predicted protein product shares 38% and 43% amino acid identity with human and mouse Cdk2, respectively. Inactivation of K03E5.3 by RNAi causes highly variable defects, with animals arresting as embryos or during various larval stages [25].

In other organisms, Cdk2 partners with A- and E-type cyclins. The functions of the *C. elegans *cyclin E gene *cye-1 *have been studied extensively. *cye-1 *mutant animals have cell division defects only during the later larval stages [51-53]. This most likely does not indicate the CYE-1 is not required earlier, but may be due to perdurance of maternal RNA or protein contribution [51,53]. The cell division defects were studied in most detail in the vulval precursor cells (VPCs), a series of 6 cells generated in late L1. Three of these undergo a series of divisions in the L3 stage to form the adult vulva. In *cye-1 *mutants, VPCs initiate division at the same time as wild-type, and undergo appropriate terminal differentiation at the normal time too. However, only 2 rounds of division take place compared to 3 in wild type. Based on *P*_*rnr*_*::GFP *expression, the delay in cell division is due to a prolonged G1 phase. Thus, *cye-1 *appears to be required for G1/S progression, similar to mammalian cyclin E-Cdk2 complexes.

RNAi for *cye-1 *causes a cell division arrest when the embryo has reached the approximately 100 cell stage [51], despite the fact that CYE-1 is expressed in all dividing cells in the embryo [53]. Three possibilities can explain this observation. First, RNAi may not fully eliminate *cye-1 *function. The fact that RNAi for *cye-1 *reduces CYE-1 protein levels below detection limits in immunostaining of early embryos argues against this possibility [53]. Second, other cyclin-CDK complexes may compensate for loss of CYE-1. Finally, the rapid early embryonic cell divisions may not require cyclin E-Cdk2 activity until the establishment of proper G1 phases. This last hypothesis does not preclude a role for cyclin A-Cdk2 complexes in early embryogenesis, a possibility that has not yet been investigated.

The results in *C. elegans *thus indicate that CYE-1, presumably complexed to a Cdk2 homolog, is essential for all cell divisions but the early embryonic divisions. An essential role for Cdk2 was also found in experiments in mammalian tissue culture and *Drosophila *[54-58]. It is surprising, therefore, that Cdk2 knockout mice were found to be viable for up to 2 years after birth [59,60], and mice lacking both E-type cyclins only show defects in late embryogenesis [61,62]. Although a recent report provides evidence that in the absence of Cdk2, cyclin E-Cdk1 complexes may regulate the G1 to S transition [63], this does not explain the relatively mild defects of cyclin E knockout mice.

### CDK-4

The *C. elegans *genome encodes only one D-type cyclin, CYD-1, and one Cdk4/6 related kinase, CDK-4, which have been shown to interact *in vitro *[64]. This greatly reduces problems of redundancy in studying these gene families. *cyd-1 *and *cdk-4 *mutant animals complete embryogenesis, but fail to initiate larval somatic blast cell divisions after hatching [64,65], although a few rounds of division of the somatic gonad precursor cells (SGPs) do occur. The arrested cells fail to express the *P*_*rnr*_*::GFP *S-phase marker, and arrest with a 2N DNA content [64,65]. Conversely, overexpression of *cyd-1*and *cdk-4 *together is sufficient to drive expression of P_*rnr*_*::GFP *[64]. Developmental processes such as cell growth and migration of the P blast cells to the ventral cord still occur [64,65], indicating that the cell-cycle arrest is not a secondary effect of interfering with development in general. Based upon these results, *C. elegans *CYD-1/CDK-4 complexes are essential for G1 to S phase progression in postembryonic cells.

RNAi of *cyd-1 *or *cdk-4 *does not block embryonic divisions [64,65]. The only effects on embryonic development were found in *cyd-1 *mutant animals, which fail to undergo the final embryonic divisions of 4 intestinal precursor cells [65] and 2 coelomocyte precursor cells [66]. Both of these cell types divide late in embryogenesis following a prolonged G1 arrest [1]. *cdk-4 *mutants do not show defects in these tissues, which could be explained if CDK-4 protein is more stable then CYD-1. Expression of CYD-1 and CDK-4 is observed starting in mid-embryogenesis, at a time when most cell divisions have completed, and is largely restricted to postproliferative lineages [64]. Thus, CYD-1/CDK-4 activity appears to be largely dispensable for embryonic cell divisions.

#### Cyclin D-Cdk4/6 targets

Whereas Cdk1 and Cdk2 likely have a multitude of targets, only two major functions have been proposed for mammalian cyclin D-Cdk4/6 complexes. First, phosphorylation of pRb by cyclin D-Cdk4/6 and cyclin E-Cdk2 inactivates pRb, and allows for expression of S-phase genes [67,68]. Second, cyclin D-Cdk4/6 complexes are thought to sequester members of the Cip/Kip family of CDK inhibitors, which may contribute to the activation of cyclin E-Cdk2 [19]. An important question that remains unanswered is the relative contribution of these mechanisms to the G1 to S transition function of cyclin D-CDKs.

*C. elegans *has a single pRb family member, LIN-35 [69], and two Cip/Kip related proteins, CKI-1 and CKI-2 (referred to as CKI-1/2 from hereon) [70]. If inactivation of LIN-35 or CKI-1/2 is an important function of CYD-1/CDK-4, then inactivating these proteins through RNAi or mutant alleles should rescue the *cyd-1 *and *cdk-4 *mutant phenotypes. Indeed, inactivation of *lin-35 *or of *cki-1/2 *rescued multiple aspects of the *cyd-1 *and *cdk-4 *phenotypes, including body size, expression of *P*_*rnr*_*::GFP *and cell division [65]. This indicates that LIN-35 and CKI-1/2 act downstream of CYD-1 and CDK-4.

Loss of *lin-35 *or *cki-1/2 *did not fully rescue the *cyd-1 *and *cdk-4 *mutant defects, and important differences in the manner of rescue were also apparent [65]. For example, loss of *lin-35 *was less proficient at restoring divisions in the P-cell lineage than loss of *cki-1/2*. In contrast, loss of *lin-35 *resulted in the normal 4 rounds of endoreplication in the intestinal nuclei, while loss of *cki-1/2 *restored only 1 round of DNA replication, likely because additional rounds of DNA replication require transcription of S-phase genes. These results indicate that *lin-35 *and *cki-1/2 *play at least partially non-overlapping roles in G1/S progression. Indeed, the effects of inactivation of *cki-1/2 *and *lin-35 *were additive in the intestinal cell lineage [65]. Whereas either alone could restore only a limited number of nuclear divisions in *cyd-1 *mutants, inactivation of both resulted in a number of divisions exceeding that in wild-type animals.

These experiments demonstrate that LIN-35 and CKI-1/2 likely act downstream of CYD-1/CDK-4 in parallel pathways. Two important questions that still need to be addressed are whether CKI-1/2 are inactivated by sequestering or through an alternative mechanism requiring CYD-1/CDK-4 kinase activity, and whether LIN-35 and CKI-1/2 are the only CYD-1/CDK-4 targets.

#### Comparisons to other organisms

*Drosophila *mutants lacking the single D-type cyclin CycD, the sole Cdk4/6 related protein Cdk4, or both, develop into viable adults, although they are smaller, have a decreased cellular growth rate, and show reduced fertility [71,72]. It appears that in *Drosophila*, CycD/Cdk4 primarily stimulate cell growth [71,73]. These results contrast with those observed in *C. elegans *where growth does not appear to be directly regulated by CYD-1 or CDK-4 [65]. More recent results indicate that *Drosophila *CycD/Cdk4 can influence G1/S progression, as Cdk4 can induce ectopic S-phase entry in the eye imaginal disk [74]. A possible contributing factor to the apparent lack of a requirement for CycD and Cdk4 is the lack of binding of Dacapo, the *Drosophila *p27 Cip/Kip family member, to CycD/Cdk4 [71]. Flies may therefore not use sequestering of Cip/Kip family members by cyclin D-Cdk4/6 complexes as a mechanisms of driving G1 progression.

Mouse embryos that lack all three D-type cyclins [75], or both Cdk4 and Cdk6 [76], die of haematopoietic abnormalities after day E13.5. Mice are thus able to undergo significant proliferation and development in the absence of cyclin D-Cdk4/6. It is possible that, in mice, cell cycle regulators show more plasticity than expected from tissue culture experiments. Other kinase complexes may take over the role of cyclin D-CDKs, including unusual combinations such as cyclin D-Cdk2. This is supported by the finding that mouse embryonic fibroblasts (MEFs) derived from these knockouts are critically dependent on Cdk2 activity for proliferation [75,76]. Alternatively, cyclin D-Cdk4/6 activity may be dispensable during rapid embryonic development, like in *C. elegans*, but play a critical role in the resumption of cell division following a prolonged period of arrest. Support for this hypothesis stems from the observation that haematopoietic stem cells from cyclin D knockout mice are severely deficient in their ability to proliferate both *in vivo *and *in vitro *[75]. Furthermore, cyclin D and CDK-4/6 knockout MEFs show defects in cell-cycle re-entry upon serum stimuation [75,76]. Future experiments using conditional knockout mice to examine the roles of D-type cyclins and Cdk4/6 in adult tissues are required to resolve these questions.

## *C. elegans *CDKs with cell-cycle independent roles

Although CDKs are best known for their role in regulating cell-cycle progression, the functions of the CDK family are not limited to this process, and include regulation of transcription, neuronal development, and other processes [77]. Cdk7, Cdk8, and Cdk9 are each implicated in the regulation of gene transcription through phosphorylation of the C-terminal domain (CTD) of RNA polymerase II [10,78,79], while CDK5 has a well described role in development of the central nervous system [80]. *C. elegans *has homologs of each of these kinases [23], but only CDK-7 and CDK-9 have been studied in detail.

### CDK-7

In higher eukaryotes, Cdk7 plays a unique dual role in regulating the activity of other CDKs as well as transcription. Cdk7 forms the kinase subunit of CAK, the CDK activating kinase responsible for the activating phosphorylation of many CDKs on a Threonine residue in the T-loop (Thr161 in human Cdk1) [10]. In addition, Cdk7 is part of the general transcription factor TFIIH, which stimulates transcription by phosphorylating the CTD of Pol II [10].

*C. elegans *has a single Cdk7 homolog, encoded by the *cdk-7 *gene [23]. *cdk-7 *mutants have dramatically reduced Pol II CTD phosphorylation, and fail to initiate transcription of all embryonic genes examined [81]. These results indicate a general requirement for CDK-7 in embryonic mRNA transcription through CTD phosphorylation.

Complete inactivation of *cdk-7 *by RNAi blocks meiosis and results in a one-cell embryonic arrest, similar to RNAi of *cdk-1 *[81]. This is consistent with CDK-7 being responsible for the activating phosphorylation of CDK-1. In addition, partial loss of *cdk-7 *function resulted in an increased cell division time [81], with prolonged mitosis as well as interphase, which indicates that CDK-7 regulates additional CDKs as well, in accordance with the role of Cdk7 in other organisms in activating multiple CDKs.

### CDK-9

In conjunction with T-type cyclins, Cdk9 forms a positive transcription elongation factor termed P-TEFb, which regulates transcriptional elongation through phosphorylation of the Pol II CTD (reviewed in [79]). Inhibition by RNAi of *C. elegans cdk-9 *or both T-type cyclins, *cyt-1.1 *and *cyt-1.2*, resulted in developmental defects similar to those caused by inactivation of essential transcription factors [82]. In addition, inactivation of *cdk-9 *or *cyt-1.1 *and *cyt-1.2 *together blocked the transcription of embryonic reporter genes [82]. The CTD is phosphorylated on two Serine residues: Ser 2 and Ser 5. Ser 5 phosphorylation is highest when Pol II is at the promoter, while Ser 2 phosphorylation is associated with the elongation step [78,83]. In accordance with Cdk9 being involved in transcriptional elongation, inactivation of *cdk-9 *or *cyt-1.1 *and *cyt-1.2 *together dramatically reduces Ser 2 phosphorylation, but not Ser 5 phosphorylation [82]. These experiments indicate that *C. elegans *P-TEFb plays a broad role in the transcription of embryonic genes.

## Developmental control of *C. elegans *cell division

During the life of *C. elegans*, environmental factors can cause a global withdrawal from cell division at two stages. First, postembryonic development is not initiated in the absence of food. Second, in conditions of limited food or overcrowding, animals can enter an alternative larval stage termed *dauer*, which is geared toward long-term survival. Entry into and exit from the dauer stage is accompanied by cessation and resumption of cell divisions. As in other model systems, developmental control of *C. elegans *cell division involves regulators of G1 progression, notably CYD-1/CDK-4, and the Cip/Kip family member CKI-1.

As mentioned above, CYD-1 and CDK-4 are required for initiation of postembryonic blast cell divisions, and overexpression of CYD-1 and CDK-4 is sufficient for entry into S-phase. However, little is known about the environmental signals that lead to activation of CYD-1/CDK-4. Similarly, the activity of CKI-1 is required for the developmental cell cycle arrest of somatic cells in starved larvae and dauer larvae, and loss of *cki-1 *by RNAi induces hyperproliferation in the embryo and multiple postembryonic cell lineages [70,84]. The CKI-1 promoter region is large and complex, and CKI-1 levels appear to be transcriptionally regulated through lineage specific transcription factors [70,85]. With respect to the L1 arrest, a recent report demonstrated a role for insulin/insulin-like growth factor signaling in regulating CKI-1 transcription [86].

In addition to transcriptional regulation, CKI-1 levels may be regulated through ubiquitin-dependent degradation [70,87]. The susceptibility of CKI-1 to degradation may be phosphorylation dependent, as the CDC-14 phosphatase has recently been shown to regulate CKI-1 activity [88].

An important question that has to be addressed is to what extent CKI-1 inactivation occurs downstream of CYD-1/CDK-4 activity, and to what extent CKI-1 can be regulated independently of CYD-1/CDK-4. Interestingly, in addition to regulating G1 progression, CKI-1 may also have a more direct function in cell fate specification. In *cki-1(RNAi) *animals a higher than normal number of distal tip cells (DTCs) (figure 2) is observed, which do not stem from duplication of DTCs but arise from other somatic gonad cells that normally do not produce DTCs, indicating a role for CKI-1 in cell-fate specification [89].

In addition to regulation of cell division by environmental cues, cell-cycle regulators can affect downstream developmental events, as described in two recent papers [31,90]. In one, CYD-1 activity is required for the asymmetric division of a precursor cell of the somatic gonad, while a second study identified a novel role for CDK-1 in coupling cell-cycle progression to cell differentiation.

### Regulation of an asymmetric division by CYD-1

In screens for abnormal gonadogenesis, a novel *cyd-1 *allele (*q626*) was identified that specifically affects the asymmetric division of the 2 somatic gonad precursor (SGP) cells, and not cell division in general [90]. In wild-type animals, the SGPs divide asymmetrically in males, and symmetrically in hermaphrodites, and express sex specific markers. In *cyd-1(q626) *males however, the SGPs divide symmetrically, and express hermaphrodite specific markers [90]. The result is a disorganized feminized gonad. CYD-1 therefore appears to regulate the asymmetric division and sex specific fate specification of the SGPs.

The *cyd-1(q626) *allele specifically affects cell fate specification of the SGPs, and is likely a partial loss-of-function *cyd-1 *allele. However, most animals with complete loss of *cyd-1 *function still undergo one or two SGP divisions, likely due to maternal contribution, and these animals show similar defects in cell-fate specification as *cyd-1(q626) *animals [90]. Further analysis showed that loss of *lin-35 Rb, efl-1 E2F, dpl-1 DP *or *cki-1,2 Cip/Kip *all suppressed the phenotype of *cyd-1(q626) *[90]. These results indicate that CYD-1 regulates the asymmetric SGP division through the pRB/E2F pathway. A candidate target of the E2F/RB pathway for regulation of SGP division is *fkh-6*, which encodes a forkhead transcription factor required for the male specific SGP division and differentiation. The *fkh-6 *promoter contains several putative E2F binding sites [90]. Expression of an FKH-6 reporter construct is abolished or delayed in *cyd-1(q626) *mutants, while loss of *efl-1 *restores transcription, indicating that EFL-1 represses *fkh-6 *transcription in wild-type animals [90]. A role for E2F in cell-fate specification of the SGPs fits with more recent findings that E2F transcription factors have roles in processes other than G1/S progression, including differentiation [91].

The *cyd-1(q626) *allele only affects the SGP divisions. Another recently isolated *cyd-1 *allele, *cyd-1(cc600)*, shows similar lineage specific defects [66]. This allele was isolated in a screen for altered numbers of coelomocytes, four of which arise from two precursor cells that divide late in embryogenesis, after a period of quiescence. In *cyd-1(cc600) *mutants, the final coelomocyte divisions do not occur, while differentiation and other cell divisions are unaffected [66]. The allele recovered is a mutation in a splice donor site, likely resulting in partial loss of function. Different tissues clearly vary in their requirement for CYD-1.

### A role for CDK-1 in coupling cell division to cell fate specification

Several *cdk-1 *alleles were identified in screens for embryonic cell fate transformation that cause an excess-endoderm phenotype, but no overt defects in cell division [31]. The observed excess-endoderm phenotype appears to be caused by stabilization of a protein called OMA-1, which normally prevents the precocious degradation of maternally provided cell fate determinants in the early embryo. Stabilization of OMA-1 causes delayed degradation of multiple cell-fate determinants, including SKN-1, PIE-1, MEX-3, and MEX-5 [92].

OMA-1 is normally degraded shortly after entry into the first mitotic division [93,94], and these experiments indicate that CDK-1 is required for the timely degradation of OMA-1. Coupling OMA-1 degradation to mitosis through the activity of CDK-1 could provide a convenient means to prevent degradation of cell fate determinants before they have been segregated into the appropriate daughter cell. For example, in the 2 cell embryo, MEX-5 is localized to the anterior blastomer, and promotes the degradation of PIE-1, which is restricted to the posterior blastomer [95,96]. Delaying degradation of OMA-1 until after mitosis may protect PIE-1 from degradation in the one cell embryo, in which MEX-5 and PIE-1 have to co-exist [31].

Regulation of OMA-1 stability by CDK-1 is likely performed in a complex with the B-type cyclin CYB-3, as RNAi for *cyb-3 *also resulted in stabilization of OMA-1 protein [31]. The *cdk-1 *mutations alter residues located in the T-loop, involved in cyclin binding and access of ATP to the kinase. Nevertheless, CDK-1 showed normal binding to CYB-1 and CYB-3, as well as CKS-1, and both cyclin B kinase complexes exhibited near wild-type Histone H1 kinase activity [31]. Thus, the effects of the mutations on CDK-1 are either very subtle, or affect the interaction of CDK-1 kinase complexes with specific substrates. No direct phosphorylation of OMA-1 by CDK-1 was observed, indicating that the effect of CDK-1 on OMA-1 is likely indirect.

In the same screen an allele of *cks-1 *was identified that also stabilizes OMA-1 protein levels. *cks-1 *is one of two *C. elegans *genes homologous to Cks/Suc1, a conserved CDK binding protein whose role(s) in cell division remain somewhat of an enigma [97]. The *cks-1 *mutation identified affects binding of CKS-1 to CDK-1, and also results in a reduction in kinase activity of CDK-1/CYB-3 towards Histone H1. These experiments uncover a potential role for *cks-1 *in modulating the inactivation of OMA-1 by CDK-1 [31].

These findings demonstrate a specific role for CDK-1 in coupling mitosis to the degradation of OMA-1, and thus to the proper asymmetric distribution of cell-fate determinants like PIE-1. They also exemplify the power of forward genetics in the identification of subtle alleles that affect only particular aspects of gene function, something that cannot be accomplished through, for example, RNA interference.

## Conclusion

As the focus in cell-cycle research shifts from unicellular organisms and cell-lines to *in vivo *research in the much more complex setting of a multicellular organism, *C. elegans *is well positioned as a model in which to study cell division during multicellular development. Already, significant novel findings have been made in *C. elegans*, including the discovery of the Cullin family of E3 ubiquitin ligase subunits [98], the developmental regulation of cell divisions through the CDC-14 phosphatase and CKI-1 [88], or the finding that CDK-1 can affect cell-fate specification [31]. In the future, research in *C. elegans *will continue to help elucidate the functioning of the cell-cycle machinery and the interplay between animal development and cell division.

## Abbreviations

MEF: Mouse Embryonic Fibroblast

DTC: Distal Tip Cell

BrdU: Bromodeoxyuridine

VPC: Ventral cord Precursor Cell

SGP: Somatic Gonad Precursor Cell
